# Large-scale (Phase III) evaluation of broflanilide 50WP (VECTRON™ T500) for indoor residual spraying for malaria vector control in Northeast Tanzania: study protocol for a two-arm, non-inferiority, cluster-randomised community trial

**DOI:** 10.1186/s12879-022-07138-3

**Published:** 2022-02-21

**Authors:** Patrick K. Tungu, Mark W. Rowland, Louisa A. Messenger, Graham J. Small, John Bradley, Janneke Snetselaar, Matthew J. Kirby, Njelembo J. Mbewe

**Affiliations:** 1 Department of Disease Control, London School of Hygiene and Tropical Medicine, London, UK; 2 Innovative Vector Control Consortium, Liverpool, UK; 3 Kilimanjaro Christian Medical University College, Moshi, Tanzania; 4 National Institute for Medical Research, Amani Research Centre, Muheza, Tanzania

**Keywords:** Insecticide resistance, Broflanilide, Clothianidin, Indoor residual spraying, Vector density, Entomological inoculation rate

## Abstract

**Background:**

Indoor residual spraying (IRS) is a major method of malaria vector control across sub-Saharan Africa. Effective control is being undermined by the rapid spread of insecticide resistance. There is major investment in development of new insecticides for IRS that possess novel modes of action, long residual activity, low mammalian toxicity and minimal cross-resistance. VECTRON™ T500, a new IRS product containing the active ingredient broflanilide as a 50% wettable powder (WP), has been shown to be efficacious against pyrethroid susceptible and resistant vector species on mud and concrete substrates in experimental hut (Phase II) trials.

**Methods:**

A two-arm non-inferiority cluster randomized controlled trial (Phase III) will be undertaken in Muheza District, Tanga Region, Tanzania. VECTRON™ T500 will be compared to the IRS product Fludora® Fusion (clothianidin 50% WP + deltamethrin 6.25% WP). The predominant malaria vectors in the study area are pyrethroid-resistant *Anopheles gambiae s.s.*, *An. arabiensis* and *An. funestus s.s*. Sixteen village clusters will be pair-matched on baseline vector densities and allocated to reference and intervention arms. Consenting households in the intervention arm will be sprayed with VECTRON™ T500 and those in the reference arm will be sprayed with Fludora® Fusion. Each month, CDC light traps will collect mosquitoes to estimate changes in vector density, indoor biting, sporozoite and entomological inoculation rates (EIR). Susceptibility to IRS active ingredients will be assessed using World Health Organisation (WHO) bottle bioassays. Target site and metabolic resistance mechanisms will be characterised among *Anopheles* field populations from both trial arms. Residual efficacy of both IRS products will be monitored for 12 months post intervention. Questionnaire and focus group discussions will explore factors that influence adherence, adverse effects and benefits of IRS.

**Discussion:**

This protocol describes a large-scale non-inferiority evaluation of a novel IRS product to reduce the density and EIR of pyrethroid-resistant *Anopheles* vectors. If VECTRON™ T500 proves non-inferior to Fludora® Fusion, it will be considered as an additional vector control product for malaria prevention and insecticide resistance management.

*Trial registration:* ClinicalTrials.gov, NCT05150808, registered on 26 November 2021. Retrospectively registered.

## Background

Vector control is an essential component of all malaria control strategies [1], with long-lasting insecticidal net (LLIN) distribution and indoor residual spraying (IRS) contributing 68% and 13%, respectively, to the estimated decline in malaria cases between 2000 and 2015 [2]. In 2019, about 253 million LLINs were delivered globally by manufacturers to malaria endemic countries and out of these, 84% were delivered to countries in sub-Saharan Africa [3]. While the provision of LLINs to endemic countries in sub-Saharan Africa increased from 145 million in 2010 to about 213 million in 2019; there has been a decline in the percentage of the population at risk protected by IRS in sub-Saharan Africa from 10.1 to 5.7% and globally from 5 to 2% during the same period [3]. The decline in IRS coverage has been attributed to the development of insecticide resistance, lack of inexpensive alternative insecticides and limited resources for annual applications of the intervention [4].

Scientific evidence of the efficacy of IRS in reducing or interrupting malaria transmission in different epidemiological settings has been available since the 1950–60s [5]. IRS is effective in controlling malaria transmission and in reducing related morbidity and mortality, provided the majority of premises within targeted communities are treated [6]. The application of IRS products consistently over time in large areas has altered the vector distribution and, subsequently, the epidemiological pattern of malaria in several African countries, including Botswana, Namibia, South Africa, eSwatini and Zimbabwe, significantly reducing major vectors of malaria, *Anopheles funestus* and *An. gambiae* sensu stricto [7]. Between 2006 and 2010, pyrethroid IRS product use was scaled up in sub-Saharan Africa mainly due to their affordability and relatively long residual effect [4]. However, development and spread of multiple mechanisms of pyrethroid resistance, including knockdown resistance mutations L1014F (kdr West) and L1014S (kdr East), across Africa poses a serious challenge to pyrethroid IRS control of malaria vectors [8]. For example, operational failure of deltamethrin IRS campaigns in South Africa was attributed to insecticide resistance observed in the malaria vector *An. funestus* [9, 10]. Similarly, the development and spread of pyrethroid resistance in *An. gambiae* [8, 9] in Equatorial Guinea and island of Bioko reduced the efficacy of IRS and LLINs [11, 12]. Mutations in the voltage-gated sodium channels, combined with metabolic resistance through overexpressed mixed function oxidase enzymes, gave clear survival advantages to their mosquito carriers [8, 13]. This necessitated the development and introduction of IRS formulations with more costly insecticides from the carbamate and organophosphate chemical classes that have a different mode of action to pyrethroids [4]. Despite the carbamate bendiocarb being highly effective, the formulated IRS product has a short residual duration, with the consequence that multiple spraying cycles are required in areas with long transmission seasons for maximum effect [4, 14]. The organophosphate pirimiphos-methyl formulated as a microcapsule suspension has shown residual duration of up to 12 months [4, 15, 16]. However, target site mutations and copy number variants have evolved in the acetylcholinesterase gene (Ace-1) that confer cross-resistance to both carbamates and organophosphates [17]. Between 2010 and 2019, resistance to carbamates and organophosphates has been detected respectively in 31.7% and 24.9% of sites that reported monitoring malaria vector resistance status [3].

The Global Plan for Insecticide Resistance Management (GPIRM) published by the WHO proposed among other things, the development of new active ingredients (AIs) with different modes of action which could be used in strategies to manage insecticide resistance [1]. These resistance management schemes are primarily based on IRS and include the use of rotations and mixtures of insecticides from different classes. Recently, IRS formulations have been developed which contain the neonicotinoid insecticide clothianidin, either as a single AI in SumiShield™ 50 WG or as a mixture with deltamethrin in Fludora® Fusion; bringing the total number of insecticide classes for IRS on the World Health Organization (WHO) Prequalification Vector Control Product Assessment Team (WHO PQT/VCP) list to four (carbamate, organophosphate, pyrethroid and neonicotinoid) [18, 19]. With IRS playing the main role in strategies for insecticide resistance management, there is a need to develop further active ingredients with new modes of action.

VECTRON™ T500, a newly developed IRS product by Mitsui Chemicals Agro, Inc. (Tokyo, Japan) has been shown to be efficacious against both pyrethroid-susceptible and -resistant mosquito species [20]. The active ingredient in VECTRON™ T500 is broflanilide, a meta-diamide *N*-[2-bromo-4-(perfluoropropan-2-yl)-6-(trifluoromethyl)phenyl]-2-fluoro-3-(*N*-methylbenzamido)benzamide] insecticide, which has a mode of action different to other insecticide classes [21]. Broflanilide is classified by the Insecticide Resistance Action Committee (IRAC) in the new class 30; GABA-gated chloride channel allosteric modulators [21, 22]. In 2021, VECTRON™ T500 demonstrated prolonged effectiveness in small-scale experimental hut trials (Phase II) against WHO Prequalified products in Tanzania and Benin [20].

For VECTRON™ T500 to be recommended by WHO PQT/VCP as a new IRS product, it must demonstrate superior or equivalent efficacy to a currently recommended WHO PQT/VCP listed IRS product in a non-inferiority community-level trial with entomological endpoints [23].

## Study objectives

### Primary objective

To determine non-inferiority of VECTRON™ T500 to the reference product Fludora® Fusion (Bayer AG, Monheim, Germany), which has been prequalified by WHO PQT/VCP, in reducing vector population density. This will be determined by the difference between the number of female *An. gambiae* sensu lato, *An. funestus s.l.* and *Culex* spp*.* caught per trap night in clusters sprayed with VECTRON™ T500 compared to Fludora® Fusion. Individuals from morphologically identical species complexes will be identified to species level using molecular diagnostics.

### Secondary objectives

- To assess the duration of residual efficacy of VECTRON™ T500 on mud and concrete sprayed wall surfaces during the 12-month post-intervention period.
- To determine seasonal sporozoite rate and entomological inoculation rates (EIRs) of vectors, and the number of *Plasmodium falciparum* infective bites received per person during the post-intervention period.
- To record the difference between the number of female *Culex* spp*.* caught per trap night in clusters sprayed with VECTRON™ T500 compared to Fludora® Fusion.
- To assess the resistance status of wild *Anopheles* and *Culex* spp. populations to any of the insecticides being used during the post-intervention period.

### Tertiary objectives

- To determine the quality of the IRS applications.
- To record adverse events among spray teams and treated households.
- To assess community acceptability of VECTRON™ T500.

## Methods/designs

### Study area and cluster selection

**Fig. 1:**
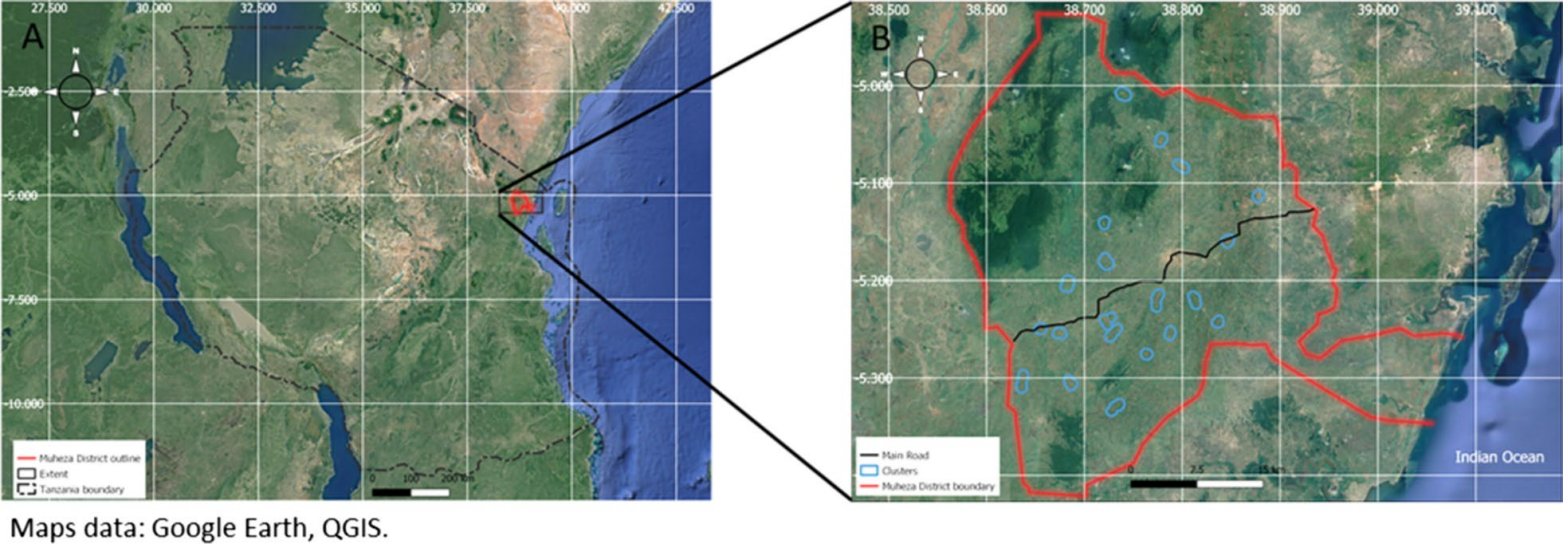
Study area **A** location of Muheza District in Northeast Tanzania; **B** proposed clusters in Muheza District. The maps of Tanzania and Muheza district were produced using QGIS 3.22 software [44] and overlaid on Google Earth satellite images by the study investigator (NJM). Using QGIS 3.22 software [44], a one kilometre transect from the mid-point to the perimeter of each village was digitized to produce the shape files for the clusters in the Muheza map

This study will be carried out in Muheza District, Tanga Region, Northeast Tanzania (Fig. 1). The population of Muheza District consists primarily of subsistence farmers [24]. The region has a tropical climate with a long rainy season from March to June and a short rainy season from October to December. Most houses within Muheza District are constructed from mud or cement with a thatched or iron roof. In 2012, surveys conducted in Muheza District found that 83% of households owned at least one ITN (insecticide-treated net), and there have been two LLIN distribution campaigns carried out since then [25]. Malaria transmission occurs all year round with two peaks in transmission following the two rainy seasons. The main malaria vectors in Muheza District are *An. gambiae* s.s., *An. arabiensis* and *An. funestus* s.s. [25]*.* Wild *An. gambiae* s.l. from Muheza District are resistant to pyrethroids [26].

### Study design

A matched cluster randomized controlled non-inferiority study design will be used as IRS in community-level intervention. The study monitoring period will be for 12 months post-intervention or beyond this date if residual efficacy on house walls indicates continuing effectiveness. Baseline data collection on vector densities will commence in the six months preceding the randomisation and pair matching of the clusters. At the onset of the long rainy season, one round of IRS will be done which will be followed by monthly vector density monitoring and *in-situ* cone bioassays. Baseline household enumeration and social-cultural and acceptability measurements will be conducted before IRS.

### Power calculation, estimation of effect size and cluster selection

Sample size calculations will be based on the study primary endpoint i.e., indoor density of *Anopheles* mosquitoes. An estimated mean of 3.0 female *Anopheles* per light trap per night will be used, referencing data from recent studies in Tanzania [27]. A paired design will be used in which clusters will be matched on baseline indoor density of *Anopheles* mosquitoes. Previous data suggests a within-pair coefficient of variation of 0.23, but a conservative value of 0.3 will be used in our calculations.

The trial is a non-inferiority design, intended to show that spraying the intervention product, VECTRON™ T500, will not result in higher malaria vector densities, by a prespecified non-inferiority margin of 50%, than those per trap per night in the reference arm sprayed with Fludora® Fusion. Using the methods described in Hayes and Moulton [28] and the assumptions listed above, it was calculated that 8 clusters per treatment arm (16 in total) will be needed for the 50% margin to have 80% power to demonstrate non-inferiority. The calculations were based on 49 trap nights per cluster. There was very limited statistical advantage in increasing trap-nights beyond 50. This number of clusters considers the loss of degrees of freedom caused by matching.

Sixteen clusters, containing 75–200 households per cluster (HHs), will be selected for spraying. Core areas of clusters should be at least 1 km apart to prevent infiltration of mosquitoes from other villages. All consenting HHs in all 16 clusters will be sprayed. Approximately 1200–3200 households will be sprayed (~ 75–200 HHs/cluster in 16 clusters).

Although a minimum of 49 households is required per cluster according to the sample size calculations, a larger hamlet will be recruited for logistical reasons, as a proportion of households may refuse to accept the IRS spraying and others may be absent during spraying or lost during follow-up.

During baseline vector density monitoring, 20 clusters will be included, to improve the pair-matching post-intervention. The four clusters with greatest variance will not be included in the post-intervention monitoring but will still receive the IRS treatment. The 16 selected clusters will be pair-matched based on vector densities and randomly allocated to the two trial arms: one arm (8 clusters) will be sprayed with the novel intervention (VECTRON™ T500) and the other arm (8 clusters), the reference product (Fludora® Fusion). All possible randomisations will be stratified on ITN coverage and wall surface to ensure minimum difference of 15% or less in mean ITN coverage and mean ratio of cement houses to mud houses per arm. From all randomisations that meet these criteria, one will be selected at random.

### Sensitisation, community and household consent

A meeting with district authorities (District Medical Officer, District Executive Director and District Commissioner) will be held at the beginning of the project to present the objectives and research activities. Meetings with community representatives and hamlet leaders will encourage adherence of inhabitants.

Community consent will be obtained from village leaders, individual verbal and written consent will be obtained from family heads and household members. All households within each community will be offered IRS.

The risks and benefits of participation will be made known and the opportunity to withdraw will be explained without loss of other benefits. Individuals already using LLINs will be encouraged to continue usage. Informed consent will be read, and consent forms signed and dated by participants. One copy of the form will be given to the household to keep for personal records, and one copy will be kept in the project files. If a participant is unable to sign the consent form, a fingerprint will be taken.

When the information is read to the family, they will be given a consent form, translated into Swahili, to read before signing. The form has been designed to give full disclosure in a format that is easy to comprehend. The language in the form is simple and aimed at a primary education level to ensure every member of the target group will be able to understand it. The field worker will be trained to ask whether the household understands all information in the form, or whether any sections need clarification.

### Enumeration of consenting households

Mapping/census of the study area will be conducted in the baseline period to update maps and add new households to the database. The census will record (a) the Global Positioning System (GPS) coordinates; (b) head of household; (c) number of occupants (adults and children); (d) number of bedrooms and total number of rooms; (e) number of sleeping places; (f) number and type of LLINs; (g) predominant wall material; and (h) predominant roof material.

Every building within each hamlet will be mapped using a GPS handheld unit (Garmin Legend e-trex) and ExpertGPS v3.8 (TopoGrafix) software. Cluster boundaries, health facilities and other landmarks will also be geo-located. Each house will be assigned a unique 8-digit household identification (ID) consisting of 3-digit village code, 2-digit hamlet code and 3-digit house code.

### Indoor residual spraying and quality control

Consenting households HHs in the intervention arm will be sprayed with VECTRON™ T500 (100 mg of AI/m^2^), those in the reference arm will receive IRS with Fludora® Fusion (200 mg/m^2^). Prospective spray operators will undergo a medical examination; those with underlying health problems will be excluded. Pregnant women will also be excluded. Spray operators recruited from the clusters will be trained according to WHO guidelines. A total of 100 spray operators will be recruited to conduct the IRS campaign, 5 from each cluster. All sleeping and living rooms in consenting houses will be sprayed, whilst kitchens and storerooms will be excluded. All walls will be sprayed and ceilings will also be sprayed, if accessible. Furniture will be removed from houses and inhabitants will be asked to remain outside during spraying and for 2–3 h afterwards.

Spray operators will be provided with personal protective equipment and clothing and health checks will be performed on them before and after spraying. If operators experience any adverse events, they will receive free medical care and the effects will be documented.

Chemical analysis of the insecticide dosages applied to the walls will be carried out as a quality control of the IRS application process. Whatman® No. 1 filter papers will be attached to the walls of HHs before spraying and then removed post-spray. Four filter papers will be used per HH on different walls, in 20 randomly selected HHs per cluster.

### Intervention monitoring

#### Vector density

CDC light traps hung inside houses next to occupied LLINs will be used to collect mosquitoes for species identification, sporozoite ELISA, and characterization of DNA-based resistance mechanisms. Four operatives will be allocated with 5 light traps each and given responsibility for trapping in 4 of the 16 clusters. In each cluster in each month there will be 3 rounds of light trap collections spaced over 3 weeks in 15 randomly selected HHs. Therefore, in each treatment arm of 8 clusters there will be 120 light trap nights in each month. All households in each cluster will be re-randomised at each monthly surveillance point. Therefore, in each cluster there will be 180 light-trap nights spread across the 12-month post intervention period, from which non-inferiority will be determined. All mosquitoes collected will be identified morphologically to genus and species complex level (*An. gambiae s.l.*, *An. funestus s.l.* and *Culex* spp.). Sub-samples taken from each cluster each month will be identified to species level using molecular diagnostic assays; numbers and proportions identified will depend on the total numbers caught.

#### Phenotypic insecticide resistance testing

WHO bottle bioassays with 1 h exposure [29] will be used to determine phenotypic resistance of wild *Anopheles* mosquitoes to broflanilide (6 µg/bottle), deltamethrin (12.5 µg/bottle) and clothianidin (90 µg/bottle). Drawing on CDC methods regarding the coating of Wheaton bottles [30], the above-mentioned diagnostic concentrations of these insecticides have been shown to kill 100% of insecticide susceptible mosquitoes. Broflanilide will be coated together with MERO 800 ppm/bottle. Insecticide solutions will be prepared from technical grade material dissolved in acetone.

*An. gambiae s.l.* and *An. funestus s.l.* will be collected as larvae from breeding sites within the study area and reared in the insectary. Progeny of these wild *An. gambiae s.l.* and *An. funestus s.l.* will then be exposed to insecticide according to standard operating procedures [29]. Knockdown will be recorded for all insecticides after 1 h and mortality will be recorded after 24 h for deltamethrin, and 72 h for broflanilide and clothianidin. Assays will be conducted at three timepoints: before IRS, at 3 months post-spraying, and at the end of the field study (12 months post-spraying) to detect whether there have been any changes in insecticide susceptibility in wild *Anopheles* populations resulting from exposure to the IRS. WHO bioassays will also be performed with *An. gambiae* s.s. Kisumu (pyrethroid-susceptible strain) and *An. gambiae* s.s. Zeneti or Muleba-Kis (pyrethroid-resistant strains) colonies for comparison and quality control.

#### Genotypic insecticide resistance monitoring

For individual mosquitoes, morphologically identified as *An. gambiae* s.l. [31], DNA will be extracted from legs/wings and used for species identification and detection of L1014S-kdr, L1014F-kdr and G119S-ace1 [17, 32, 33]. Sporozoite detection (CSP-ELISA) will be performed on a subset of PCR-confirmed *An. gambiae* s.s. and *An. arabiensis* [34]. Separate pools of 5 individuals, PCR-confirmed as either *An. gambiae* s.s. or *An. arabiensis*, will be prepared and RNA extracted. cDNA will be synthesized and metabolic gene expression measured relative to susceptible *An. gambiae* s.s. and *An. arabiensis* colony strains; CYP6M2, CYP6P3, CYP6P4, CYP66Z3, CYP9J5 and CYP9K1 will be measured in *An. gambiae* s.s. and CYP6AA1, CYP6AA2, CYP6P15P, CYP6P1, CYP6AD1, COE_4679, CYP6P3, CYP6P4 and 15,786 (ester hydrolase) in *An. arabiensis* [35].

For individual mosquitoes, morphologically identified as *An. funestus* s.l. [31], DNA will be extracted from legs/wings and used for species identification and detection of L119F-GSTe2, CYP6P9a and CYP6P9b. Sporozoite detection (CSP-ELISA) will be performed on a subset of PCR-confirmed *An. funestus* s.s. Separate pools of 5 individuals, PCR-confirmed as *An. funestus* s.s., will be prepared and RNA extracted [31, 36–39]. cDNA will be synthesized and metabolic gene expression measured relative to a susceptible *An. funestus* s.s. colony strain; CYP6N1, CYP6M7, CYP6M1 and CYP6Z3 will be measured [36].

#### IRS residual activity

WHO cone bioassays on IRS treated walls of houses will be used to assess residual activity in both trial arms at monthly intervals until the end of the trial 12 month post-IRS intervention [40]. Plastic WHO cones will be fixed to four walls per house placed in a diagonal arrangement across the room, and one additional cone will be fixed to an outside untreated wall to act as a control. Of the four selected households per cluster, two will be constructed out of cement and the other two out of mud, to compare substrate materials. To determine IRS product efficacy both pyrethroid susceptible and pyrethroid resistant strains will be used. In each cone test, 10 *An. gambiae* s.s. mosquitoes [40] will be aspirated into each cone and exposed for 30 min. After exposure, mosquitoes will be held in paper cups with cotton wool soaked in 10% glucose solution. Immediate knockdown and delayed mortality at 24, 48 and 72-h will be scored. Susceptible and resistant mosquitoes will be tested in the same room on alternate days according to availability. The pyrethroid resistant *An. gambiae* s.s. Zeneti strain possesses the L1014S-kdr mutation and elevated oxidases levels conferring metabolic resistance. If wild *An. gambiae* s.s. are in short supply, adult F1 of *An. funestus* s.l. or *An. gambiae* s.s. Muleba-kis will be laboratory-reared for monthly residual bioassays. With 4 test houses per cluster, and 4 cone tests per house, up to 160 susceptible and 160 resistant mosquitoes will be tested per cluster per month.

### Operational safety and community acceptance

#### Safety

The health status of spray men will be examined before and after spraying by qualified medical staff. Spray men will be asked to complete a questionnaire immediately after spraying has finished and then one month later to determine what, if any, adverse events they may have experienced and at what frequency. Similar questionnaires on adverse events will also be distributed in the community after spraying. Study participants will also be encouraged to contact members of the study team if they experience health issues that they believe are related to the IRS.

An independent IRS human safety assessment for VECTRON™ T500 will be conducted by Environmental Resources Management (ERM) according to the terms of the ‘WHO Generic risk assessment model for indoor residual spraying of insecticides' [41]. According to the risk assessment report, the insecticide is non-mutagenic and non-genotoxic, shows low levels of mammalian toxicity, and at the application rate proposed (100 mg/m^2^) poses acceptable risk of exposure to professional spray operators and to adult residents sleeping in treated houses; this being about 30-fold and 100-fold lower than the acceptable daily intake, respectively, using the WHO model [42].

#### Acceptability

Questionnaires on the ease of insecticide application will be given to all spray operators while questionnaires on community acceptability will be given to a random sample of two clusters per study arm immediately post-spraying and then repeated with the same sub-sample after 6 months.

Focus group discussions (FGDs) will be carried out with a subsample of community participants (8 participants in each FGD) in two randomly selected clusters in each arm. These will be performed six months post-intervention. Within these discussions, factors such as perceived adverse events, benefits, supplementary usage of nets and other means of mosquito protection will be explored and discussed.

### Data analysis

Data analysis will be carried out using NVivo software for qualitative data and Stata (Version 16) for quantitative data. For the FGD data, emerging themes will be assessed using the NVivo software. Count and binary data will be analysed using Generalised Linear Models. Data analysis will be carried out using cluster level summaries since random effects models perform poorly in a matched design with fewer than 20 clusters per arm [28]. For the primary endpoint of mosquito density, the ratio of densities in each matched pair will be calculated and inference will be based on a paired *t*-test. Adjustment will be made for time post-spraying, LLIN use and wall type using the two-stage method described by Hayes and Moulton [28]. The secondary outcomes of EIR, sporozoite rate, estimated number of infective bites per person per year, and residual bioefficacy will be analysed in the same way.

### Data management

Household data collected during the census and during entomological data monthly surveillance will be captured in electronic forms on smartphones installed with Open Data Kit (ODK) collect. The data will be stored on a secure server located at PAMVERC (Pan African malaria vector research consortium) at NIMR/KCMC/LSHTM. All data management and analyses will be done using Stata software.

Standard operating procedures (SOP) for data collection will be developed and field study staff trained. QC will be conducted by a supervisor who will monitor field staff for completeness and internal consistency of responses. Data collected on paper forms (mosquito identification, insecticide resistance results) will be double entered by two data clerks.

All data will be uploaded into a secure server. Data will be stored encrypted and access to the data will be restricted only to authorised study investigators and data management staff. A unique identifier number will be given to each household to safeguard confidentiality.

Upon completion of the study, electronic files will be stored on a server and also copied to encrypted USB. Electronic data and paper source records will be retained for a minimum of 10 years.

### Oversight and monitoring

A Trial Steering Committee (TSC) will be established to provide study oversight. The TSC members will be independent of the trial and its institutions and have the necessary expertise to monitor study progress and participant safety. Although the risk to study participants is considered minimal from either of the study IRS products, their safety experiences will be documented.

### Ethics, informed content and dissemination

This study has been approved by the ethical review committee of the Ministry of Health in Tanzania (NIMR/HQ/R.8a/VOL.IX/3520), the institutional review board of LSHTM (N° 22,459). The study is registered on clinicaltrials.gov (NCT05150808, registered on 26 November 2021). The study will be conducted according to the Declaration of Helsinki and the International Guidelines for Ethical Review of Epidemiological Studies. The protocol of the study has been reviewed by the independent Expert Scientific Advisory Committee of the IVCC and by the WHO Prequalification Vector Control Team (WHO PQT/VCT).

Before any project activities, villages and hamlet leaders and local health staff will be invited to sensitisation sessions. Community health workers within each cluster will be fully informed as to the aims of the study and will be on hand to answer day-to-day questions. The consent form will be in Swahili and indicate the purpose of the study, procedures, risks, and benefits of the intervention. All participation is voluntary.

Findings of the study will be shared with international policymakers such as WHO PQT/VCT and WHO Vector Control Advisory Group (VCAG). The project will involve the engagement of national, local, and community authorities and leaders. The findings will be published in peer reviewed journals and shared with the product manufacturer, project funders and presented at the annual meeting of the WHO Roll Back Malaria Vector Control Working Group.

## Discussion

Development of new IRS products containing insecticides whose efficacies are not compromised by pre-existing cross-resistance in vector populations is necessary for three reasons: for reinvigorating malaria vector control, for sustaining malaria transmission gains achieved, and for providing opportunity for insecticide resistance management. Establishment of non-inferiority of these new IRS products to WHO PQT/VCP prequalified standards in cluster randomised trials will provide predictive evidence for their community-level performance in the control of malaria vectors against a backdrop of LLIN universal coverage. This protocol describes a non-inferiority two-arm cluster randomised trial of the impact of VECTRON ™ T500 compared to Fludora® Fusion on changes in malaria vector densities and EIR, and evaluates their residual activities.

Since LLINs are the primary means of malaria prevention in Tanzania, it is likely that families within the communities will be using nets or other vector control tools to protect themselves. IRS is a community level intervention; the presence of other vector control tools such as LLINs in families in the two trial arms may affect the ability to measure the relative contribution of the IRS products to reduce vector densities. However, it should be possible to stratify the analysis within each cluster to assess whether there is any additional effect or interaction from secondary vector control or personal protection measures on the effect of IRS [27]. The matched pair allocation of IRS to intervention and reference arms in this trial will take into account such variation of secondary vector control tool usage at community level during random assignment of IRS treatments to clusters.

While the main intervention for malaria prevention in Africa remains standard LLINs, LLINs containing a pyrethroid and piperonyl butoxide are increasing their market share, and there is also increasing interest in dual active ingredient LLINs [43]. Although the proportion of the sector that includes IRS has declined overall [4], there is increasing recognition by the Global Fund that next generation LLINs will not be sufficient by themselves by themselves to eliminate malaria; there is need to reconsider the value of next generation LLINs in combination with new forms of IRS in public health initiatives such as the Net Transition Initiative of the Global Fund. Interventions like VECTRON™ T500 and other recent IRS products will be essential to achieving the aim of country or regional malaria elimination.

## Data Availability

All relevant data, the full protocol, and informed consent forms are available upon reasonable request from the principal investigator, Professor Mark Rowland.

## Abbreviations

CDC: Centers for Disease Control and Prevention
cDNA: Complementary deoxyribonucleic acid
CSP-ELISA: Circumsporozoite protein enzyme linked immunosorbent assay
DNA: Deoxyribonucleic acid
EIR: Entomological inoculation rates
ERM: Environmental resource management
FGD: Focus group discussion
GABA: Gamma aminobutyric acid
GPIRM: Global Plan for Insecticide Resistance Management
GPS: Global positioning system
HH: Household
IRS: Indoor residual spraying
ITN: Insecticide treated net
kdr: Knock down resistance
LLIN: Insecticidal long-lasting net
NMCP: National malaria control programme
PAMVERC: Pan African Malaria Vector Control Consortium
PCR: Polymerase chain reaction
RNA: Ribonucleic acid
s.s: Sensu stricto
WG: Water-dispersible granules
WHO: World Health Organisation

## References

[1] World Health Organization. Global plan for insecticide resistance management in malaria vectors. 2012. https://apps.who.int/iris/handle/10665/44846.

[2] Bhatt S, Weiss DJ, Cameron E, Bisanzio D, Mappin B, Dalrymple U (2015). The effect of malaria control on *Plasmodium falciparu*m in Africa between 2000 and 2015. Nature.

[3] World Health Organization. World Malaria Report 2020: 20 years of global progress and challenges. Geneva, Switzerland: World Health Organisation; 2020. https://apps.who.int/iris/handle/10665/337660.

[4] Oxborough RM (2016). Trends in US President’s Malaria Initiative-funded indoor residual spray coverage and insecticide choice in sub-Saharan Africa (2008–2015): Urgent need for affordable, long-lasting insecticides. Malar J.

[5] MacDonald G (1957). The epidemiology and control of malaria.

[6] Pluess B, Tanser FC, Lengeler C, Sharp BL (2010). Indoor residual spraying for preventing malaria. Cochrane Database Syst Rev.

[7] Sharp BL, Kleinschmidt I, Streat E, Maharaj R, Barnes KI, Durrheim DN (2007). Seven years of regional malaria control collaboration—Mozambique, South Africa, and Swaziland. Am J Trop Med Hyg.

[8] Kleinschmidt I, Rowland M, Koenraadt CJM, Spitzen J, Takken W (2021). Insecticides and malaria. Innovative strategies for vector control-Ecology and control of vector-borne diseases.

[9] Hargreaves K, Koekemoer LL, Brooke BD, Hunt RH, Mthembu J, Coetzee M (2000). Anopheles funestus resistant to pyrethroid insecticides in South Africa. Med Vet Entomol.

[10] Hargreaves K, Hunt RH, Brooke BD, Mthembu J, Weeto MM, Awolola TS (2003). Anopheles arabiensis and An. quadriannulatus resistance to DDT in South Africa. Med Vet Entomol.

[11] Curtis GF, Miller JE, Hodjati MH, Kolaczinski JH, Kasumba I (1998). Can anything be done to maintain the effectiveness of pyrethroid-impregnated bednets against malaria vectors?. Philos Trans R Soc B Biol Sci.

[12] Clarke SE, Bøgh C, Brown RC, Pinder M, Walraven GEL, Lindsay SW (2001). Do untreated bednets protect against malaria?. Trans R Soc Trop Med Hyg.

[13] Kolaczinski JH, Fanello C, Hervé J-P, Conway DJ, Carnevale P, Curtis CF (2000). Experimental and molecular genetic analysis of the impact of pyrethroid and non-pyrethroid insecticide impregnated bednets for mosquito control in an area of pyrethroid resistance. Bull Entomol Res.

[14] Bradley J, Matias A, Schwabe C, Vargas D, Monti F, Nseng G (2012). Increased risks of malaria due to limited residual life of insecticide and outdoor biting versus protection by combined use of nets and indoor residual spraying on Bioko Island, Equatorial Guinea. Malar J.

[15] Oxborough RM, Kitau J, Jones R, Feston E, Matowo J, Mosha FW (2014). Long-lasting control of Anopheles arabiensis by a single spray application of micro-encapsulated pirimiphos-methyl (Actellic® 300 CS). Malar J.

[16] Rowland M, Boko P, Odjo A, Asidi A, Akogbeto M, N’Guessan R (2013). A new long-lasting indoor residual formulation of the organophosphate insecticide Pirimiphos methyl for prolonged control of pyrethroid-resistant mosquitoes: an experimental hut trial in Benin. PLoS ONE.

[17] Weill M, Malcolm C, Chandre F, Mogensen K, Berthomieu A, Marquine M (2004). The unique mutation in ace-1 giving high insecticide resistance is easily detectable in mosquito vectors. Insect Mol Biol.

[18] Oxborough RM, Seyoum A, Yihdego Y, Dabire R, Gnanguenon V, Wat’Senga F (2019). Susceptibility testing of Anopheles malaria vectors with the neonicotinoid insecticide clothianidin; results from 16 African countries, in preparation for indoor residual spraying with new insecticide formulations. Malar J.

[19] WHO. List of WHO prequalified Vector Control Products. 2020. https://extranet.who.int/pqweb/vector-control-products/prequalified-product-list. Accessed 1 Nov 2021.

[20] Snetselaar J, Rowland MW, Manunda BJ, Kisengwa EM, Small GJ, Malone DJ (2021). Efficacy of indoor residual spraying with broflanilide (TENEBENAL), a novel meta-diamide insecticide, against pyrethroid- resistant anopheline vectors in northern Tanzania: an experimental hut trial. PLoS ONE.

[21] Nakao T, Banba S (2016). Broflanilide: a meta-diamide insecticide with a novel mode of action. Bioorg Med Chem.

[22] IRAC. IRAC Mode of Action Classification Scheme. IRAC; 2021. https://irac-online.org/mode-of-action/.

[23] World Health Organization. Data requirements and protocol for determining non-inferiority of insecticide-treated net and indoor residual spraying products within an established WHO policy class (WHO/CDS/GMP/2018.22.Rev.1.). 2019. https://apps.who.int/iris/handle/10665/276038.

[24] National Bureau of Statistics. 2012 Population and housing census. Dar es Salaam, Tanzania.: National Bureau of Statistics; 2012.

[25] Mtove G, Mugasa JP, Messenger LA, Malima RC, Mangesho P, Magogo F (2016). The effectiveness of non-pyrethroid insecticide-treated durable wall lining to control malaria in rural Tanzania: study protocol for a two-armed cluster randomized trial. BMC Public Health.

[26] Kabula B, Tungu P, Malima R, Rowland M, Minja J, Wililo R (2014). Distribution and spread of pyrethroid and DDT resistance among the Anopheles gambiae complex in Tanzania. Med Vet Entomol.

[27] Protopopoff N, Mosha JF, Lukole E, Charlwood JD, Wright A, Mwalimu CD (2018). Effectiveness of a long-lasting piperonyl butoxide-treated insecticidal net and indoor residual spray interventions, separately and together, against malaria transmitted by pyrethroid-resistant mosquitoes: a cluster, randomised controlled, two-by-two fact. Lancet.

[28] Hayes RJ, Moulton LH (2017). Cluster randomised trials.

[29] WHO. Test procedures for insecticide resistance monitoring in malaria vector mosquitoes. 2nd edn. Geneva, Switzerland: WHO; 2018. http://www.who.int/malaria/publications/atoz/9789241511575/en/.

[30] CDC. Guideline for evaluating insecticide resistance in vectors using the CDC bottle bioassay. Atlanta: CDC; 2010. https://www.cdc.gov/malaria/resources/pdf/fsp/ir_manual/ir_cdc_bioassay_en.pdf.

[31] Gillies MT, De Meillon B. The Anophelinae of Africa South of the Sahara (Ethiopian Zoogeographical Region). 2nd edn. Johannesburg: South African Institute for Medical Research; 1968.

[32] Bass C, Nikou D, Donnelly MJ, Williamson MS, Ranson H, Ball A (2007). Detection of knockdown resistance (kdr) mutations in Anopheles gambiae: a comparison of two new high-throughput assays with existing methods. Malar J.

[33] Bass C, Williamson MS, Field LM (2008). Development of a multiplex real-time PCR assay for identification of members of the Anopheles gambiae species complex. Acta Trop.

[34] Burkot TR, Williams JL, Schneider I (1984). Identification of *Plasmodium falciparum*-infected mosquitoes by a double antibody enzyme-linked immunosorbent assay. Am J Trop Med Hyg.

[35] Matowo J, Weetman D, Pignatell P, Wright A, Charlwood JD, Kaaya R, et al. Insecticide resistance characteristic of Anopheles vector species successfully controlled by deployment of pyrethroid and PBO long lasting insecticidal treated nets in Tanzania. bioRxiv. 2021. https://www.biorxiv.org/content/10.1101/2021.03.19.436139v1.

[36] Vezenegho SB, Bass C, Puinean M, Williamson MS, Field LM, Coetzee M (2009). Development of multiplex real-time PCR assays for identification of members of the Anopheles funestus species group. Malar J.

[37] Riveron JM, Yunta C, Ibrahim SS, Djouaka R, Irving H, Menze BD (2014). A single mutation in the GSTe2 gene allows tracking of metabolically based insecticide resistance in a major malaria vector. Genome Biol.

[38] Weedall GD, Mugenzi LMJ, Menze BD, Tchouakui M, Ibrahim SS, Amvongo-Adjia N (2019). A cytochrome P450 allele confers pyrethroid resistance on a major African malaria vector, reducing insecticide-treated bednet efficacy. Sci Transl Med..

[39] Mugenzi LMJ, Menze BD, Tchouakui M, Wondji MJ, Irving H, Tchoupo M (2019). Cis-regulatory CYP6P9b P450 variants associated with loss of insecticide-treated bed net efficacy against Anopheles funestus. Nat Commun.

[40] WHO. Guidelines for testing mosquito adulticides for indoor residual spraying and treatment of mosquito nets. Geneva, Switzerland: WHO; 2006. http://apps.who.int/iris/bitstream/handle/10665/69296/WHO_CDS_NTD_WHOPES_GCDPP_2006.3_eng.pdf;sequence=1.

[41] WHO. Generic risk assessment model risk model for indoor residual spraying of insecticides. 2nd edn. Geneva: WHO; 2018. https://www.who.int/publications/i/item/9789241513753.

[42] Rose P, Tenebenal TM. IRS human safety assessment for submission to the LSHTM and national ethics committees. Report NO: 0387820-TOX2. (Unpublished). 2020.

[43] Rowland M, Protopopoff N (2018). Dawn of the PBO-pyrethroid long lasting net—light at last. Outlooks Pest Manag.

[44] QGIS Development Team. QGIS Geographic Information System. 2021. http://qgis.osgeo.org.

